# Physical activity and sedentary behavior in patients with Systemic Lupus Erythematosus

**DOI:** 10.1371/journal.pone.0193728

**Published:** 2018-03-05

**Authors:** Domenico Paolo Emanuele Margiotta, Fabio Basta, Giulio Dolcini, Veronica Batani, Marina Lo Vullo, Alessia Vernuccio, Luca Navarini, Antonella Afeltra

**Affiliations:** Unit of Allergology, Clinical Immunology and Rheumatology, Università Campus Bio-Medico di Roma, Rome, Italy; National Institute of Health, ITALY

## Abstract

**Introduction:**

The aim of this study was to evaluate the proportion of patients with Systemic Lupus Erythematosus (SLE) who did not met the WHO recommendations for physical activity and to evaluate the amount of time spent in sedentary behavior.

**Methods:**

SLE patients were consecutively enrolled in a cross sectional study. The type and the time spent in physical activity and sedentary behavior were evaluated using the IPAQ short form questionnaire. The adequate physical activity was defined according to the 2010 WHO recommendations for health and the sedentary behavior according to the 2017 SBRN consensus. We also assessed quality of life using SF-36, mood disorders using BDI and HAM-H, fatigue using Facit-Fatigue and sleep disorders using PSQI scores.

**Results:**

Physical activity was not sufficient to meet WHO recommendations in 56 of 93 SLE patients (60%). SLE patients spent a median (95% range) of 180 (0–600) minutes everyday in sedentary activities. The length of daily sedentary time was more than 6 hours in 25% of SLE patients. In multivariable analysis, the factors associated to the probability of not meeting WHO criteria was only the time of exposure to antimalarials (OR 0.88, p 0.03) and the factors related to the probability of being in the upper tertile of sedentary time (more than 270 minutes) were age (OR 1.04, p 0.02), disease activity expressed by SELENA-SLEDAI score (OR 1.2, p 0.01) and Facit-fatigue score (OR 0.94, p 0.04).

**Conclusion:**

A relevant proportion of SLE patients were inadequately physically active. It is essential to improve the awareness of the importance of increase physical activity and reduce sedentary time. A better control of disease activity and fatigue and a prolonged use of antimalarials could help to reach this notable goal.

## Introduction

Physical activity has been recognized as a pivotal component of health since ancient times. In 400 B.C., Hippocrates, the Greek pioneer of modern medicine, said that: “if there is any deficiency in food or exercise the body will fall sick”. Physical activity is associated to a reduction of risk of cardiovascular diseases (CVDs), coronary hearth diseases (CHDs) and stroke [1]. It has been shown that a cardiovascular risk reduction occurs at levels of 150 minutes of at least moderate-intensity activity per week [2, 3]. Moreover, a similar weekly activity is able to decrease the risk of diabetes mellitus and metabolic syndrome [4]. Nevertheless, the beneficial effects of physical activity are not limited to cardio-metabolic health but extend to have less vertebral and hip fractures and to the prevention of breast and colon cancer [2]. Finally, physical activity is a strong protective factor against mood disorder, in particular, depression [2].

Large epidemiologic studies demonstrated that physical activity is associated to a risk reduction in incidence of several systemic chronic inflammatory conditions such as Rheumatoid Arthritis, Multiple Sclerosis and Inflammatory Bowel Diseases [5].

On the opposite side of the spectrum of physical activity, physical inactivity is one of the most serious health problems worldwide [6]. Physical inactivity is the fourth lading risk factor for global mortality, following arterial hypertension, tobacco use and hyperglycemia [2]. Large epidemiological data indicate that physical inactivity could be the principal risk factor for almost 27% of diabetes cases and 30% of CHDs case [2]. Despite the clear epidemiological significance, there is still no agreement on the definition of physical inactivity, in particular, in its sedentary behavior component [7, 8]. Epidemiological data clearly demonstrated that sedentary behavior is a risk factor for cardio-vascular, metabolic and cancer-related mortality and morbidity [9–11]. Moreover, the increase in the epidemiological risk conferred by the sedentary behavior is independent of the regular execution of physical activity, even intense [8, 10]. In the past decades, referring to the international and national recommendations for the promotion of physical activity, the sedentary behavior was considered as the absence of adequate physical activity. In other words, a person with no moderate- to-vigorous physical activity (MVPA), suggested by the WHO or other recommendations, was considered “sedentary” [12]. Today, a sedentary behavior is considered an entity distinct from the lack of adequate physical activity. In fact, the sedentary behavior involves maintaining a sitting position [12]. The introduction of the concept of MET (metabolic equivalent of task) provided an indispensable basis for the scientific definition of a sedentary behavior. The last definition proposed by the The Sedentary Behavior Research Network (SBRN) is: “Sedentary behavior refers to any waking behavior characterized by an energy expenditure ≤1.5 MET-minute while in a sitting or reclining posture” [13]. The sedentary behavior extends to many activities of our daily life and includes domestic activities (as TV viewing, talking on the phone, reading, bathing, eating, ect.), work-school activity (as Computer work, writing, etc.), transportation (as driving or riding in a vehicle) and leisure (as playing an instrument, arts and crafts, knitting/sewing, playing cards or board games, etc.) [12, 13].

Several evidences suggest a reduction of physical activity and a considerable prevalence of physical inactivity in patients affected by Systemic Lupus Erythematosus (SLE) [5, 14, 15]. SLE is a complex autoimmune disease with a possible involvement of multiple organs including skin and mucosae, kidneys, central and peripheral nervous system, cardiovascular system, bone and peripheral blood and many others [16]. SLE patients present a markedly increased risk of incidence and mortality due to CVDs [17]. Moreover, a high prevalence of Metabolic Syndrome (MeS) has been described in SLE [18–22]. The need to control cardio-metabolic risk makes it essential to promote adequate physical activity in SLE patients. The actual WHO global recommendations on physical activity for health require 150 minutes of moderate-to-vigorous-intensity physical activity per week or 75 minutes of vigorous-intensity activity per week [2].

The aim of this study was to evaluate the prevalence of physical activity, physical inactivity and the amount of sedentary behavior in SLE patients and to describe the factors related to physical inactivity and sedentary behavior in this autoimmune disease.

## Methods

### Study population

Patients affected by SLE according to SLICC classification criteria [23] were consecutively enrolled at University Campus Bio-Medico outpatient clinic between January 2015 and December 2015. All enrolled patients were continuously followed in our Lupus Clinic from diagnosis until the enrollment in this study. The possibility to take part in the study was orally proposed during outpatient outreach visits. Exclusion criteria for SLE patients were: recent pregnancy (<2 years before enrollment), active malignancy, end-stage lupus nephritis, treatment with Belimumab in the last two years, previous diagnosis of mood disorders or ongoing therapy for mood disorders.

### Sample size calculation

For sample size calculation and power analysis we considered data concerning physical inactivity and low weekly exercise previously reported in SLE patients [24, 25]. Setting a significance level of 0.05 (alpha), power at 80% (beta), an Expected proportion of 0.3, and a total wight of confidence intervals of 0.2, we estimated a saple size of 88 subjects to assess the confidence intervals for proportion. Sample size calculation and power analysis were performed using SAS University Edition, SAS Institute Inc., SAS Campus Drive, Cary, North Carolina 27513, USA.

### Ethical considerations

Ethics committee of Università Campus Bio-Medico di Roma approved the study, which complied with the Declaration of Helsinki. All the study participants provided signed an informed consent prior to enrolment.

### Clinical evaluation of SLE patients

We used the Safety of Estrogens in Lupus Erythematosus National Assessment disease activity index (SELENA-SLEDAI) to assess lupus disease activity [26, 27]. Disease flares were expressed using SELENA-SLEDAI Flares Index (SFI) [28]. Damage accrual was evaluated by the SLICC damage index (SDI) [29]. Therapy exposure was assessed, in particular, cumulative exposure to glucocorticoids, antimalarials and immunosuppressants. We used a cut off of 7.5 mg of prednisone or equivalents to define high daily dose glucocorticoid regimens, according to the evidences that link this cut off dose to an increased risk of damage accrual, in particular on cardio-metabolic damages [30]. The presence of arterial hypertension, low high density lipoprotein (HDL) cholesterol, high triglycerides levels, impaired fasting glucose or diabetes mellitus, overweight and Metabolic Syndrome were defined according to International Federation of Diabetes (IFD) criteria for MeS [31].

### Evaluation physical activity

We used the International Physical Activity Questionnaire (IPAQ) short form [32]. IPAQ short form is composed of four domains: vigorous physical activities, moderate physical activities, walking, sitting activities. The questionnaire provides for the evaluation of the number of weekly days in which physical activity is carried out and its average daily duration. The questionnaire refers to the physical activity of the last week. The IPAQ questionnaire provides both a categorical and a continuous score. However, given the non-normal distribution of energy expenditure in many populations, the continuous score is presented as median minutes or median MET-minutes. Median values can be computed using the following formulas:

- Walking MET-minutes/week = 3.3 * walking minutes * walking days;
- Moderate MET-minutes/week = 4.0 * moderate-intensity activity minutes * moderate days;
- Vigorous MET-minutes/week = 8.0 * vigorous-intensity activity minutes * vigorous-intensity days.

A combined total physical activity MET-min/week can be computed as the sum of Walking + Moderate + Vigorous MET-min/week scores. The values of MET-min for the different intensity of physical activities were previously reported [33, 34].

The categorical score is a three levels variable:

1. *Category 1*: *INACTIVE*. This is the lowest level of physical activity. Those individuals who not meet criteria for Categories 2 or 3 are considered inactive.
2. *Category 2*: *MINIMALLY ACTIVE*. The minimum pattern of activity to be classified as.sufficiently active. is any one of the following 3 criteria: (a) 3 or more days of vigorous activity of at least 20 minutes per day OR (b) 5 or more days of moderate-intensity activity or walking of at least 30 minutes per day OR (c) 5 or more days of any combination of walking, moderate-intensity or vigorous intensity activities achieving a minimum of at least 600 MET-min/week.
3. *Category 3*. *HEPA ACTIVE*. Patient active according to Health Enhancing Physical Activity (HEPA) standards. This category requires almost one of the following criteria: (a) vigorous-intensity activity on at least 3 days achieving a minimum of at least 1500 MET-minutes/week OR (b) 7 or more days of any combination of walking, moderate-intensity or vigorous intensity activities achieving a minimum of at least 3000 MET-minutes/week.

Using the data of the IPAQ questionnaire we also derived the following variables:

- Total Minutes per week of vigorous physical activity
- Total Minutes per week of moderate to vigorous physical activity (MVPA)
- Number of patients who meet the 2010 WHO recommendations of physical activity for health [2]
- Total minutes per day of sedentary activities (defined as any waking behavior characterized by an energy expenditure ≤1.5 MET-minute while in a sitting or reclining posture) [13].

### Evaluation of Health-related Quality of Life, fatigue, mood disorders and sleep quality

We evaluated the following factor associated to Health-related Quality of Life (QoL), using validated questionnaires: the Italian version of Medical Outcomes Study (MOS) 36-Items Short-Form Healthy Survey (SF-36) [35], the Italian version of The Functional Assessment of Chronic Illness Therapy (FACIT)–Fatigue [36]. Moreover, depressive symptoms were quantified by the Beck Depression inventory (BDI version II) [37] and anxiety symptoms were evaluated by Hamilton Anxiety rating scale (HAM-H) [38]. The Alexithymia construct was evaluated by Toronto Alexithymia Scale (TAS-20) [39]. The sleep quality was assessed using the Pittsburgh Sleep Quality Index (PSQI) [40].

### Statistical analysis

We analyzed the following continuous variables: Total weekly MET-min, vigorous activity weekly MET-min, moderate activity weekly MET-min, walking weekly MET-min. We used the following binary variable:—WHO physically active or inactive (patients who met or not WHO recommendation).

A validated cut-off for time spent in sedentary behavior has not been defined. So we analyzed the time spent in sedentary behavior as multinomial variable using the cut-off derived from tertiles distribution of the variable.

To evaluate the impact of possible confounders we used a linear MIXED regression model to estimate the least squared means and to compare the average of two variables. The confounders taken into account in the different models were: age, education level, alexithymia construct, disease duration.

The variables significantly associated to the physical inactivity or sedentary behavior in univariate analysis were added to the multivariable models. To analyze the variable associated to physical inactivity we used a generalized linear model (SAS PROC GENMOD) with binomial distribution of dependent variable (physical inactivity, yes or no) and logit link function to create a multivariable logistic regression model. A generalized linear regression model (SAS PROC GENMOD) with multinomial distribution of dependent variable and cumulative logit link function was built to analyze the variables associated to time spent in sedentary behavior (odds ratio to be in upper tertile of time in sedentary behavior vs lower ones).

Significance level adopted was two tailed p< 0.05. All statistical analyses were performed with SAS University Edition, SAS Institute Inc., SAS Campus Drive, Cary, North Carolina 27513, USA.

## Results

### Demographic, cardio-metabolic and SLE disease features of enrolled subjects

We enrolled 93 SLE patients, 6 male and 87 female. In Table 1 were reported demographic and SLE disease features of the enrolled sample. The mean age ± standard deviation of SLE patients was 47.3 ± 13.7 years, with average disease duration of 9.8 ± 6.8 years.

**Table 1:** Demographics and lupus disease features.

Demographic Features	Number, N	93
	Sex, M/F, N (%)	6/87 (7%/93%)
	Age, years, mean ± SD	47.3 ± 13.7
	Disease duration, years, mean ± SD	9.8 ± 6.8
Disease component (according to BILAG: almost 1 BILAG from A to C)	Neuro-Psychiatric disease, N (%)	25 (27)
	Active Renal disease, N (%)	10 (11)
	Costitutional disease, N (%)	68 (73)
	Muscoloskeletal disease, N (%)	39 (42)
	Cardiovascular and respiratory disease, N (%)	14 (15)
	Hematologic disease, N (%)	25 (27)
Previous Involvement (BILAG D)	Previous Renal disease, N (%)	19 (20)
Anti-phospholipid Syndrome		19 (20)
SLE therapy	Mean monthly actual glucocorticoids dosage, mg of prednisone or equivalents, mean ± SD	139.2 ± 129.4
	Cumulative exposure to glucocorticoids, years, mean ± SD	7.9 ± 6.8
	Cumulative exposure to glucocorticoids, percentage of disease duration, mean ± SD	78% ± 34%
	Cumulative exposure to high dose glucocorticoids (daily dose ≥ 7.5 mg), years, mean ± SD	3.8 ± 5.5
	Cumulative exposure to high dose glucocorticoids (daily dose ≥ 7.5 mg), percentage of disease duration, mean ± SD	39% ± 41%
	Antimalarial, ongoing, N (%)	64 (68)
	Antimalarial, cumulative exposure, years, mean ± SD	4.9 ± 4.4
	Antimalarial, cumulative exposure, percentage of disease duration, mean ± SD	61% ± 39%
	Azathioprine, N (%)	33 (35)
	Methotrexate, N (%)	15 (16)
	Mycophenolate Mofetil, N (%)	11 (12)
	Oral Cyclophosphamide, N (%)	4 (4)
	IV Cyclophosphamide (in the last 1 year), N (%)	5 (5)
	Other immunosuppressant, N (%)	8 (9)
	Rituximab in the last 2 years, N (%)	9 (10)
	Belimumab in the last 2 years, N (%)	0 (0)
Disease Activity and Damage	Actual SELENA-SLEDAI, mean ± SD	3.6 ± 4.2
	Mean SELENA-SLEDAI last year, mean ± SD	4.5 ± 5.0
	Mean number of flares, last 12 months, mean ± SD	0.5 ± 0.9
	SDI, mean ± SD	0.6 ± 0.8
Traditional Cardio-vascular and metabolic risk factors (according to IFD criteria)	Metabolic Syndrome, N (%)	31 (33)
	Obesity (waist circumference ≥ 80 cm for women and ≥ 94 com for men), N (%)	44 (47)
	Raised triglycerides level ≥ 150 mg/dL or specific treatment for this lipid abnormality, N (%)	21 (23)
	Reduced HDL cholesterol < 50mg/dL in females, or specific treatment for this lipid abnormality, N (%)	25 (27)
	Raised blood pressure systolic BP ≥ 130 or diastolic BP ≥ 85 mm Hg, or treatment of previously diagnosed hypertension, N (%)	41 (44)
	Raised fasting plasma glucose (FPG) ≥ 100 mg/dL or previously diagnosed type 2 diabetes, N (%)	8 (9)
	Current Smokers, N (%)	14 (15)
	CVD personal history, N (%)	10 (11)

Abbreviations: M, male; F, female; IV, intra-venous; BILAG, British Isles Lupus Assessment Group [41]

### Physical activity in SLE patients

Considering categorical IPAQ score, we found that 42 patients (44%) were physically inactive (category 1), 32 patients (34%) were minimally active (category 2) and 20 patients (22%) were in the HEPA category of physical activity.

We found that 37 SLE patients (40%) met the WHO recommendation of physical activity. The physical activity of the remaining 56 patients (60%) was not sufficient to meet WHO recommendation.

Thirty-four SLE patients (92%) who met WHO recommendations were physically active according to IPAQ. However, 18 patients (32%), physically active according to IPAQ, did not meet the WHO recommendations.

We reported in Table 2 the results of IPAQ analyzed as continuous score. The median (95% range) value of Minutes / week of vigorous activity was 0 (0–240), with a median MET-min/week value of 0 (0–1440). SLE patients performed moderate physical activity for a median of 60 (0–840) minutes / week, with a median energy expenditure of 240 (0–3360) MET-min/week. Considering walking activities, the median minutes / week of physical activity was 70 (0–450), with a median MET-min /week of 225 (0–1500).

**Table 2:** Results of IPAQ as continuous score.

Type of Physical Activity	Variable	Median (95% range)
Vigorous Activity	Days / week	0 (6)
	Minutes / day	0 (90)
	Minutes / week	0 (240)
	MET-min / week	0 (1440)
Moderate Activity	Days / week	2 (7)
	Minutes / day	30 (180)
	Minutes / week	60 (840)
	MET-min / week	240 (3360)
Walking	Days / week	3 (7)
	Minutes / day	25 (120)
	Minutes / week	70 (450)
	MET-min / week	225 (1500)

Abbreviations: MET-min, metabolic equivalent of task-minute.

### Sedentary behavior in SLE patients

SLE patients spent a median (95% range) of 180 (0–600) minutes / day in sedentary activities (sedentary behavior). Considering the variable minutes / day spent in sedentary activities, the value of the 33^rd^ percentile was 145 minutes and the value of the 66^th^ percentile was 270 minutes. Twenty-five percent of SLE patients spent more than 360 minutes / day in sedentary behavior. Ten percent of SLE reported a daily sedentary time longer that 520 minutes.

Among SLE patients who met WHO recommendations, 15 (43%) were in the lowest tertile of sedentary time (time in sedentary behavior less than 145 minutes /day), another 14 (40%) patients were in the middle tertile and 6 patients (17%) were in the upper tertile (time in sedentary behavior > 270 minutes /day), p = 0.008, Table 3.

**Table 3:** Relation between sedentary behavior and physical activity.

	Time in Sedentary Behavior (Tertiles)			
Meeting WHO recom	Lower Tertile (<145 min/d), N (%)	Middle Tertile (145–270 min/d), N (%)	Highest Tertile (>270 min/d), N (%)	TOTAL
No, N (%)	14 (25)	14 (25)	27 (50)	55
Yes, N (%)	15 (43)	14 (40)	6 (17)	35
TOTAL	29	28	33	93

The table analyzes the relation between the time spent in sedentary behavior and the compliance with WHO recommendation. The compliance with WHO recommendation was expressed as binary variable (yes or no), the time spent in sedentary behavior was stratified in three levels (distribution of the variable in tertiles). Table Fisher exact test: P 0.008. Data in parenthesis express row percentages. Abbreviations: min/d, minutes/day; recom, recommendations.

### Demographic, quality of life and SLE related features according to physical activity and sedentary behavior

No differences in age, disease duration, time of exposure to glucocorticoids, number of recent disease flares and actual SDI score were found in patients who met and who did not met WHO recommendation for physical activity. In the model adjusted for age and disease duration, we found that patients who meet WHO recommendation had a significantly longer time of exposure to antimalarials (p = 0.0003), lower disease activity score (actual SELENA-SLEDAI score) (p = 0.04), and higher score in the physical component summary (MCS) of SF-36 (p = 0.014). However, in the fully adjusted model (age, disease duration, education level and alexithymia score) the difference in MCS was no longer significant. The complete results of the analysis were reported in Table 4.

**Table 4:** Demographic, quality of life and SLE related features according to physical activity.

Parameter	Class	Mean (SE) Model I	p>t	Mean (SE) Model II	p>t	Mean (SE) Model III	p>t
Age, years	WHO inactive	47.8 (1.8)	0.68				
	WHO active	46.6 (2.3)					
Disease Duration, years	WHO inactive	10.1 (0.9)	0.68				
	WHO active	9.5 (1.1)					
Actual SELENA-SLEDAI	WHO inactive	5.25 (0.67)	0.06	5.67 (1.22)	0.04		
	WHO active	3.30 (0.82)		3.54 (1.12)			
Number of flares, last year	WHO inactive	0.57 (0.12)	0.13	0.57 (0.12)	0.14		
	WHO active	0.28 (0.15)		0.28 (0.15)			
Actual SDI	WHO inactive	0.64 (0.11)	0.29	0.26 (0.10)	0.36		
	WHO active	0.45 (0.13)		0.48 (0.12)			
Cumulative exposure to GC, years	WHO inactive	7.7 (0.9)	0.69	7.7 (0.4)	0.61		
	WHO active	8.2 (1.1)		8.1 (0.5)			
Cumulative exposure to hd GC, years	WHO inactive	3.8 (0.8)	0.99	3.7 (0.7)	0.65		
	WHO active	3.8 (0.9)		4.1 (0.8)			
Cumulative exposure to antimalarial, years	WHO inactive	4.0 (0.6)	0.01	3.9 (0.5)	0.003		
	WHO active	6.4 (0.7)		6.5 (0.6)			
BDI score	WHO inactive	12.8 (1.22)	0.12	12.6 (1.1)	0.12	12.0 (1.01)	0.44
	WHO active	9.7 (1.50)		9.7 (1.5)		10.80 (1.23)	
HAM-H score	WHO inactive	15.92 (1.35)	0.05	15.9 (1.3)	0.05	15.33 (1.24)	0.19
	WHO active	11.79 (1.62)		11.8 (1.5)		12.79 (1.49)	
Facit-Fatigue	WHO inactive	31.46 (1.43)	0.071	31.54 (1.38)	0.07		
	WHO active	35.59 (1.76)		35.47 (1.69)			
PSQI score	WHO inactive	7.78 (0.59)	0.13	7.75 (0.58)	0.14		
	WHO active	6.33 (0.73)		6.38 (0.71)			
SF-36 MCS	WHO inactive	48.6 (3.0)	0.11	48.7 (3.0)	0.13	51.1 (2.80)	0.53
	WHO active	56.1 (3.7)		55.9 (3.6)		53.8 (3.38)	
SF-36 PCS	WHO inactive	43.6 (2.8)	0.011	43.7 (2.7)	0.014	45.5 (2.8)	0.09
	WHO active	54.9 (3.4)		54.7 (3.3)		53.2 (3.4)	

The models compares the least square mean values in patients physically inactive according to WHO (patients who did not met WHO recommendations) versus patients who met WHO recommendations. The least square means were estimated using Linear Mixed Models. Model I: an adjusted. Model II: adjusted for age and disease duration. Model III: adjusted for age, disease duration, education level and TAS score. Abbreviations: SE, standard error. MeS+, patients with MeS. MeS-, patients without MeS. GC, glucocorticoids. hd GC, high doses of glucocorticoids (daily dose ≥ 7.5 mg of prednisone or equivalents). MCS, mental component summary. PCS, physical component summary.

We reported in Table 5 the analysis of SLE disease features in relation to physical activity and sedentary behavior. We found that the majority of SLE patients with musculoskeletal, neuropsychiatric and active renal involvement according BILAG did not meet WHO recommendation and were in the upper tertiles of sedentary time, with significant results only for the neuropsychiatric involvement.

**Table 5:** Disease domain (BILAG classification) according to physical activity and sedentary behavior.

	Physically Active according to WHO criteria			Time spent in Sedentary Behavior			
	No	Yes	p	<145 min/d	145–270 min/d	>270 min/d	p
Constitutional Involvement, N (%)	41 (60)	27 (40)	0.7	22 (33)	21 (31)	25 (36)	0.7
Muscolo-skeletal involvement, N (%)	22 (57)	17 (43)	0.8	8 (19)	11 (29)	20 (52)	0.1
Active Renal Disease, N (%)	8 (80)	2 (20)	0.2	1 (10)	4 (40)	5 (50)	0.2
Neuro-psychiatric involvement, N (%)	15 (60)	10 (40)	0.9	4 (16)	6 (24)	15 (60)	0.01
Cardio-Pulmonary involvement, N (%)	10 (67)	5 (33)	0.5	3 (20)	5 (33)	7 (47)	0.3

Abbreviations: min/d, minutes/day.

The prevalence of arterial hypertension (27 (66%) vs 14 (34%) p = 0.3), reduced HDL cholesterol (18 (72%) vs 7 (28%) p = 0.15), increased triglycerides levels (13 (62%) vs 8 (38%) p = 0.8), impaired fasting glucose or type 2 diabetes (7 (87%) vs 1 (13%) p = 0.09) and overweight (30 (68%) vs 14 (32%) p = 0.1) did not significantly differ between patients according to meeting or not WHO recommendations. Metabolic Syndrome was diagnosed in 23 (74%) SLE patients who did not met WHO recommendation and in 8 (26%) of patients physically active according to WHO criteria.

SLE patients were stratified in two classes according to time spent in Sedentary Behavior: patients in the lowest and middle tertiles versus patients in the upper tertile of time in Sedentary Behavior. We found that patients in the upper tertile of sedentary behavior were older (p = 0.009), presented a higher disease activity score (p<0.0001) and a greater number of recent disease flares (p = 0.004) compared to patients in the lowest tertiles. Moreover, the score of depressive symptoms BDI (p = 0.01), the score of anxiety symptoms HAM-H (p = 0.002) and the score of sleep disorders PSQI (p = 0.01) were increased in patients in the upper tertile of sedentary time. Patients in the upper tertiale of sedentary time presented reduced score of the mental (p = 0.012) and physical (p = 0.0004) component summary of SF-36. However, the differences in BDI, HAM, MCS and PCS scores between subjects in upper and lowers tertiles of sedentary behavior were less significant in the fully adjusted model. Complete results were reported in Table 6.

**Table 6:** Demographic, quality of life and SLE related features according to sedentary behavior.

Parameter	Class: Time in Sedentary Behavior	Mean (SE) Model I	p>t	Mean (SE) Model II	p>t	Mean (SE) Model III	p>t
Age, years	Lowest tertiles	44.6 (1.7)	0.009				
	Highest tertile	52.3 (2.3)					
Disease Duration, years	Lowest tertiles	9.4 (0.9)	0.43				
	Highest tertile	10.6 (1.2)					
Actual SELENA-SLEDAI	Lowest tertiles	2.3 (0.5)	<0.0001	2.2 (0.5)	<0.0001		
	Highest tertile	6.1 (0.7)		6.2 (0.7)			
Number of flares, last year	Lowest tertiles	0.25 (0.11)	0.004	0.26 (0.11)	0.008		
	Highest tertile	0.81 (0.1)		0.80 (0.16)			
Actual SDI	Lowest tertiles	0.45 (0.10)	0.058	0.49 (0.10)	0.19		
	Highest tertile	0.78 (0.14)		0.72 (0.13)			
Cumulative exposure to GC, years	Lowest tertiles	7.13 (0.9)	0.16	7.5 (0.4)	0.11		
	Highest tertile	9.2 (1.2)		8.6 (0.6)			
Cumulative exposure to hd GC, years	Lowest tertiles	3.3 (0.7)	0.20	3.4 (0.6)	0.27		
	Highest tertile	4.9 (1.0)		4.6 (0.9)			
Cumulative exposure to antimalarial, years	Lowest tertiles	5.3 (0.6)	0.38	5.4 (0.5)	0.23		
	Highest tertile	4.4 (0.7)		4.3 (0.7)			
BDI score	Lowest tertiles	9.8 (1.1)	0.01	10.2 (1.1)	0.07	11.1 (0.9)	0.46
	Highest tertile	14.6 (1.6)		13.8 (1.6)		12.4 (1.3)	
HAM-H score	Lowest tertiles	11.9 (1.2)	0.002	12.4 (1.2)	0.01	12.9 (1.2)	0.07
	Highest tertile	18.5 (1.7)		17.7 (1.7)		16.6 (1.6)	
Facit-Fatigue	Lowest tertiles	36.6 (1.3)	0.0002	35.7 (1.3)	0.001		
	Highest tertile	27.6 (1.7)		28.4 (1.8)			
PSQI score	Lowest tertiles	6.3 (0.6)	0.01	6.5 (0.6)	0.04		
	Highest tertile	8.7 (0.7)		8.4 (0.7)			
SF-36 MCS	Lowest tertiles	55.9 (2.8)	0.012	55.4 (2.9)	0.03	54.9 (2.7)	0.10
	Highest tertile	43.7 (3.8)		44.6 (4.0)		47.2 (3.7)	
SF-36 PCS	Lowest tertiles	53.7 (2.6)	0.0004	53.1 (2.6)	0.002	52.7 (2.7)	0.01
	Highest tertile	37.6 (3.5)		38.8 (3.6)		41.1 (3.7)	

The models compares the least square mean values of variables in patients in the lower and middle tertiles of time spent in Sedentary Behavior versus patients in the upper tertile of time in Sedentary behavior. The least square means were estimated using Linear Mixed Models. Model I: an adjusted. Model II: adjusted for age and disease duration. Model III: adjusted for age, disease duration, education level and TAS score. Abbreviations: SE, standard error. MeS+, patients with MeS. MeS-, patients without MeS. GC, glucocorticoids. hd GC, high doses of glucocorticoids (daily dose ≥ 7.5 mg of prednisone or equivalents). MCS, mental component summary. PCS, physical component summary.

Considering the distribution in tertiles of sedentary time, Metabolic Syndrome was diagnosed in 6 (19%) SLE patients in the lower tertile, in 10 (32%) patients in the middle tertile and in 15 (49%) in the upper tertile (p = 0.02).

In the correlation analysis, the values of total MET-min/week negatively correlated with age (r -0.3, p 0.01), actual SELENA-SLEDAI score (r -0.24, p 0.02), the number of last year flares (r -0.28, p 0.007), the BDI score (-0.35, p 0.0008), the HAM-H score (r -0.39, p 0.0002). Conversely, the total MET-min/week was in direct correlation with the exposure time to antimalarials (r 0.27, p 0.007).

The total time spent in sedentary behavior directly correlated with age (r 0.4, p 0.0001), actual SELENA-SLEDAI (r 0.32, p 0.001), last values of damage index (SDI) (r 0.26, p 0.01), the number of recent disease flares (r 0.3, p 0.005), the score of depressive symptoms BDI (r 0.5, p <0.0001), the score of anxiety symptoms HAM-H score (r 0.5, p<0.0001), the score of sleep disorders PSQI (r 0.4, p 0.0009). The total time of sedentary behavior negatively correlated with the mental (r -0.4, p 0.0001) and physical (r -0.5, p<0.0001) component summary of SF-36 and with the Facit-Fatigue score (r -0.5, p<0.0001).

We reported in Table 7 the results of multivariable logistic models analyzing the variables associated to the probability of being physically inactive according to WHO and to be in the upper tertile of sedentary time. The variables included in the model predicting the probability of being physically inactive according to WHO were cumulative time of exposure to antimalatials, actual SELENA-SLEDAI score, the score of anxiety symptoms, the facit-fatigue, and the physical component summary of SF-36. The model predictive of sedentary time was built as a multinomial logistic regression including as independent variables age, actual SELENA-SLEDAI score, the number of recent flares, the Facit-fatigue score, the score of anxiety symptoms, the mental and the physical component summary of SF-36.

**Table 7:** Multivariable analysis of the factors associated to physical inactivity and to sedentary behavior.

	Dependent Variable:			
	Physical Inactivity: not meeting WHO criteria (binomial distribution) Reference level: physically active (meeting WHO criteria)		Upper tertile of time in Sedentary Behavior (multinomial distribution) Reference level: lowest tertiles of sedentary time	
Independent Variables	OR (95% CI)	p	OR (95% CI)	p
Age, years			1.04 (1.01–1.07)	0.02
Cumulative exposure to antimalarials, years	0.88 (0.78–0.99)	0.03		
Actual SELENA-SLEDAI	1.03 (0.91–1.16)	0.62	1.2 (1.04–1.38)	0.01
Actual SDI				
Number of disease flares last year			0.92 (0.50–1.69)	0.79
HAM score	1.01 (0.93–1.08)	0.89	0.97 (0.9–1.04)	0.41
Facit Fatigue score	0.99 (0.93–1.06)	0.85	0.94 (0.88–0.99)	0.04
PCS score	0.98 (0.95–1.01)	0.20	1.01 (0.96–1.03)	0.97
MCS score			0.98 (0.95–1.02)	0.48

We reported in the table the results of the multiple logistic regression models. The first model evaluates the predictors of the probability of not meeting WHO recommendations. The second model is a multinomial logistic analysis evaluating the predictors of the probability of being in the upper tertile of time spent in sedentary behavior. Variables with significantly associated to the WHO physical inactivity or to sedentary in univariate analysis were added to the multivariable models. Abbreviations: OR, odds ratio. 95% CI, 95% confidence intervals.

## Discussion

In the present study we evaluated the prevalence of physical inactivity and sedentary behavior in SLE patients. We found that 60% of SLE patients perform physical activity below what is recommended by WHO. The average weekly time of moderate-to vigorous activity (MVPA) in our SLE sample was about 60 minutes, very far away from what is required by WHO. Using the IPAQ categorical analysis, the proportion of SLE patients classified as inactive was 44%. However, according to IPAQ score only 22 (20%) SLE patients were in the upper physical activity category reaching the HEPA standards. Overall, these data suggest that about two-third of SLE patients are insufficiently physically active or completely inactive.

According to the national survey by the Italian Ministry of Health’s Behavioral Risk Factor Surveillance System (Progressi delle Aziende Sanitarie per la Salute in Italia (PASSI), conducted by the National Centre for Epidemiology, Surveillance, and Health Promotion, 36% of Italian adults (aged 30–60 years) met the recommended physical activity levels. The prevalence of physical activity meeting WHO recommendation in the female sample of the survey was 37% [42]. Notably, the mean age of the sample was 44.7 years and was similar to the mean age of the SLE sample enrolled in our study. The sub-analysis of the PASS project, published in 2017, showed that about 32.2% of the Rome inhabitants did not meet the WHO recommendations for health [43].

We did not perform a direct comparison of physical activity between SLE and healthy subjects. However, our SLE sample and the subjects enrolled in the last PASSI survey live in Rome, sharing several epidemiological and cultural features.

Our data are in concert with what reported by Mancuso et al. 2011. The study by Mancuso et al. analyzed physical activity in 50 SLE patients using the Paffenbarger Physical Activity and Exercise test. The authors reported a mean weekly time spent in moderate-to-vigorous activity (MVPA) of 132 minutes and a proportion of patients who did not meet WHO recommendation of 72% [25].

We also analyzed the time spent in sedentary behavior in SLE patients. About one-third of our SLE sample spends more than 4.5 hours everyday in sedentary activities. Moreover, almost one in four SLE patients reported a sedentary daily time longer than 6 hours. Three recent studies reported that sedentary daily time seems not to significantly differ between SLE patients and healthy controls [44–46]. However, the experimental design of the three studies and the features of enrolled SLE samples were different. In the study of Eriksson et al. an extensive questionnaire on physical activity was used, while in the studies by Legge et al. and Pinto et al. the time spent in physical activity and in sedentary behavior was estimated using accelerometry. We could not evaluate the sedentary time of our SLE sample in comparison to a reference healthy population. However, large epidemiological data clearly demonstrated that sedentary time is associated to an increased hazard ratio of all-cause mortality, cardiovascular disease incidence and mortality, type 2 diabetes mellitus incidence and cancer incidence and mortality [10]. In the majority of the prospective studies the cut-off of sedentary time associated to the increased risk of adverse outcome was 6–10 hours per day.

Several berries to physical activity were described in SLE patients. These impediments included muscolo-skeletal manifestations (arthralgias, arthritis), cardio-pulmonary involvement, mood disorders as depression, fatigue and fibromyalgia [14, 25]. We found that the majority of SLE patients who did not meet WHO recommendations or who spent long time in sedentary behavior presented muscolo-skeletal involvement, but the results did not reach statistical significance. These results could be due to the low prevalence of musculoskeletal manifestations in our SLE sample.

In our analysis, the patients who did not meet WHO recommendations for physical activity seem to present a more active SLE disease, a reduced time of exposure to antimalarials, more severe symptoms of mood disorders and a reduce score of physical component of QoL. Moreover, these variables were inversely correlated to the total weekly MET-min, an assessment of energy expenditure. The possible impact of mood disorders on physical exercise in SLE has already been described. Mancuso et al. reported a reduced physical activity in patients with more social stress and more fatigue [25]. The association of physical activity and physical component of SF-36 could be an expression of pain (the bodily pain component of SF-36), physical functioning, role physical and general health perception. However, in the multivariable analysis, after controlling the model for possible confounders, the only factor that seems to be associated to not meeting the WHO criteria was the exposure to antimalarials. In particular, every year of continuative exposure to antimalarials reduces by 12% the probability of being physically inactive. Antimalarials are a pivotal part of SLE long-term therapy, helping to improve disease management [47]. Literature data and clinical practice clearly demonstrate the impact of antimalarials on disease activity, disease flare and damage accrual [48, 49]. Moreover, antimalarials are the only non-biological therapies with a positive impact on fatigue and SLE related quality of life [50, 51]. Antimalarials are useful treatment in muscolo-skeletal SLE manifestations. Finally, several data, including one our recent study, demonstrated a possible cardio-metabolic protective effect of antimalarials [17, 21]. These multiple therapeutic effects could explain the association that we found between physical activity and the length of exposure to antimalarials.

In this study we also analyzed the factors associated to the time spent in sedentary behavior among SLE patients. In univariable analysis, patients who spent longer time in sedentary behavior presented older age, increased SLE disease activity and a major number of recent flares. Moreover, patients with prolonged sedentary habit presented more fatigue, more severe depressive and anxiety symptoms and reduced scores in both mental and physical components of SF-36. The possible impact of mood disorders on sedentary behavior has been described in general population [52, 53]. However, in SLE, scientific data on possible determinants of sedentary behavior are still missing. In the multivariable model, we found that the principal factors associated to increased sedentary time could be age, SLE disease activity and fatigue. Several data from clinical trials demonstrated that physical exercise could improve fatigue in SLE patients [14]. We hypnotize that the relation between sedentary time and fatigue could be a vicious circle, in which patients with severe fatigue tend to be sedentary; on the other hand, a longer sedentary time, along with a reduced physical activity, could contribute to the worsening of fatigue.

Our study presents several limitations. First of all, the cross-sectional design did not allow the inference of causality relations. Furthermore, the sample size did not make it possible to stratify patients according to the type of immunosuppressant used. Likewise, we could not categorize the musculoskeletal domain analyzing separately arthralgias and/or myalgias from more severe manifestation as arthritis. Another limitation was the instrument we used to measure physical activity and sedentary behavior. The use of IPAQ implies that the type of physical activity and the time spent in the different type of exercise were self-reported by patients. In SLE patients the results of self reported variables could be affected by several factors. For this reason, we adjusted the analysis for confounders as education levels, alexithymia, mental and physical components of SF-36. Finally, we could not extrapolate the screen sedentary time (television time, computer time and others type of screen time) from IPAQ short form. Several data suggest that the screen time is particularly associated to adverse outcomes [10].

In conclusion, we found that a relevant proportion of our SLE sample, about 60% of patients, did not met WHO recommendations on physical activity. Furthermore, one in four SLE patients spent more then 6 hours everyday in sedentary behavior. We found a prolonged daily sedentary time also in about one in five patients physically active according to WHO criteria. Both inadequate physical activity and sedentary behavior are independent risk factors for mortality and morbidity, mainly from cardiovascular diseases and cancer. In SLE patients the promotion of adequate physical activity and the reduction of sedentary time is particularly relevant, given the severely and precociously increased cardiovascular risk. Our data suggest that we have to improve the awareness of SLE patients and healthcare professionals on the pivotal role for health of increasing physical activity and reducing the sedentary time. According to out finding, a proper control of SLE disease activity and an improved management of fatigue, along with a more effective patient education, could contribute to fight against sedentary behavior. The prolonged use of antimalarials could play a role in facilitating the practice of adequate physical exercise.
